# A multicentre double-blind randomised controlled trial evaluating the efficacy of daily use of antibacterial mouthwash against oropharyngeal gonorrhoea among men who have sex with men: the OMEGA (Oral Mouthwash use to Eradicate GonorrhoeA) study protocol

**DOI:** 10.1186/s12879-017-2541-3

**Published:** 2017-06-28

**Authors:** Eric P. F. Chow, Sandra Walker, Jane S. Hocking, Catriona S. Bradshaw, Marcus Y. Chen, Sepehr N. Tabrizi, Benjamin P. Howden, Matthew G. Law, Kate Maddaford, Tim R. H. Read, David A. Lewis, David M. Whiley, Lei Zhang, Andrew E. Grulich, John M. Kaldor, Vincent J. Cornelisse, Samuel Phillips, Basil Donovan, Anna M. McNulty, David J. Templeton, Norman Roth, Richard Moore, Christopher K. Fairley

**Affiliations:** 1 Melbourne Sexual Health Centre, Alfred Health, 580 Swanston Street, Carlton, VIC 3053 Australia; 2 Central Clinical School, Faculty of Medicine, Nursing and Health Sciences, Monash University, Melbourne, VIC 3004 Australia; 3 Centre for Epidemiology and Biostatistics, Melbourne School of Population and Global Health, The University of Melbourne, Melbourne, VIC 3053 Australia; 4 Department of Microbiology and Infectious Diseases, The Royal Women’s Hospital, Parkville, VIC 3052 Australia; 5 Murdoch Childrens Research Institute, Parkville, VIC 3052 Australia; 6 Department of Obstetrics and Gynaecology, The University of Melbourne, Parkville, VIC 3052 Australia; 7 Microbiological Diagnostic Unit Public Health Laboratory, Department of Microbiology and Immunology, The University of Melbourne, Peter Doherty Institute for Infection and Immunity, Parkville, VIC 3010 Australia; 8 Western Sydney Sexual Health Centre, Western Sydney Local Health District, Parramatta, NSW 2150 Australia; 9 Marie Bashir Institute for Infectious Diseases and Biosecurity & Sydney Medical School-Westmead, The University of Sydney, Westmead, NSW 2145 Australia; 10 Pathology Queensland Central Laboratory, QLD, Brisbane, 4029 Australia; 11 The University of Queensland Centre for Clinical Research, Royal Brisbane and Women’s Hospital Campus, QLD, Herston, 4029 Australia; 12 Prahran Market Clinic, Prahran, VIC 3181 Australia; 13 The Kirby Institute, UNSW Sydney, Kensington, NSW 2052 Australia; 14 Sydney Sexual Health Centre, Sydney Hospital, Sydney, NSW 2000 Australia; 15 School of Public Health and Community Medicine, UNSW Sydney, Kensington, NSW 2052 Australia; 16 RPA Sexual Health, Community Health, Sydney Local Health District, Camperdown, NSW 2050 Australia; 17 Central Clinical School, The University of Sydney, Camperdown, NSW 2006 Australia; 18 Northside Clinic, Fitzroy North, VIC 3068 Australia

**Keywords:** Men who have sex with men, Sexually transmitted infection, Gonorrhoea, Mouthwash, Prevention, Oropharyngeal, Throat, Topical antiseptics, Prophylaxis

## Abstract

**Background:**

Gonorrhoea is one of the most common sexually transmissible infections in men who have sex with men (MSM). Gonorrhoea rates have increased substantially in recent years. There is concern that increasing gonorrhoea prevalence will increase the likelihood of worsening antibiotic resistance in *Neisseria gonorrhoeae*. A recent randomised controlled trial (RCT) demonstrated that a single-dose of mouthwash has an inhibitory effect against oropharyngeal gonorrhoea. We are conducting the first RCT to evaluate whether daily use of mouthwash could reduce the risk of acquiring oropharyngeal gonorrhoea.

**Methods/design:**

The OMEGA (Oral Mouthwash use to Eradicate GonorrhoeA) study is a double-blind RCT and will be conducted at several sexual health clinics and high caseload General Practice (GP) clinics in Melbourne and Sydney, Australia. A total of 504 MSM attending the participating sites will be recruited. Participants will be randomised to either using ‘Study mouthwash A’ or ‘Study mouthwash B’ for 12 weeks. Study mouthwash A was inhibitory against *N. gonorrhoeae* in vitro, whereas study mouthwash B was not. Participants will be instructed to rinse and gargle the study mouthwash for 60 seconds every day. The primary outcome is the proportion of participants with oropharyngeal gonorrhoea detected by nucleic acid amplification test by 12 weeks.

**Discussion:**

The results from this trial may provide a novel way to reduce gonorrhoea prevalence and transmission without the use of antibiotics that may be associated with development of resistance. If shown to be effective, the widespread use of mouthwash will reduce the prevalence of oropharyngeal gonorrhoea, which plays key role in driving the emergence of gonococcal antimicrobial resistance through DNA exchange with oral commensal bacteria. The anticipated net effect will be interruption of onward transmission of *N. gonorrhoeae* within high density sexual networks within MSM populations.

**Trial registration:**

Australian New Zealand Clinical Trials Registry ACTRN12616000247471, registered on 23rd February 2016.

## Background

*Neisseria gonorrhoeae* is the causative agent of one of the most common sexually transmissible infections (STIs) worldwide and in recent years has increased substantially in Australia [1] and in other high income countries [2–4]. It is estimated that there were 78 million new cases of gonorrhoea among individuals aged 15–45 years globally in 2012, with over 55% in males [5].

Australian national surveillance data show that the gonorrhoea notification rate in men was stable between 2006 and 2009 (around 50 cases per 100,000 male population). However, rates have increased substantially since 2010 reaching 117 cases per 100,000 male population in 2015 [1] with the majority of cases occurring in men who have sex with men (MSM). Rises in other STIs such as syphilis and increasing rates of previously uncommon diseases such as lymphogranuloma venereum (LGV) are further evidence of the overall rising STI trend among MSM [6–10]. In addition, STIs can also increase the risk of HIV acquisition [11–13]. Biomedical interventions for HIV such as pre-exposure prophylaxis (PrEP) and ‘Treatment as Prevention’ (TasP) have been shown to reduce the risk of HIV transmission [14–18]. However, these interventions have been associated with an increase in the number of sexual partners and a decrease in condom use for anal sex, practices associated with increased risk of STIs [15, 19–21]. With the scale up of PrEP in Australia and elsewhere [22, 23], STIs including gonorrhoea are likely to rise further [24].

The rising number of gonorrhoea cases is of particular concern because gonorrhoea is becoming more difficult to treat [25]. Gonorrhoea has become resistant to almost all previously recommended treatments and successful treatment currently relies on use of the last main class of antimicrobial agents suitable for first-line therapy, specifically extended spectrum cephalosporins such as ceftriaxone [26]. To reduce the emergence of further resistance, ceftriaxone is now frequently given with a second antibiotic, typically single-dose azithromycin, as dual therapy [27–29]. Extensively antibiotic-resistant *N. gonorrhoeae* isolates have been reported in a few countries in recent years but these strains not yet spread widely [30–36]. As there are very limited options for treating these strains of *N. gonorrhoeae* [37–40], *N. gonorrhoeae* is considered as an ‘urgent resistance threat’ by the US Centers for Disease Control and Prevention (CDC) [41], a ‘priority organism’ in the Australian National Antimicrobial Resistance Strategy [42] and a ‘global threat’ by the World Health Organization (WHO) [43, 44]. One key strategic objective of the WHO Global Action Plan [45] to prevent the emergence of multidrug and cephalosporin-resistant gonorrhoea is to reduce the prevalence of infection, particularly in the oropharynx where resistance is thought to be developed via spontaneous mutation as well as genetic exchange of resistance genes with commensal *Neisseria spp.* cohabiting the same environment [46, 47].

To reduce the prevalence of gonorrhoea, it is important to understand the transmission dynamics and main drivers of gonorrhoea incidence in MSM. One might assume that gonorrhoea incidence is largely driven by penile-anal sex [48], in a similar manner to chlamydia [49]. However, unlike chlamydia, where infection is mostly asymptomatic [50, 51], urethral gonorrhoea is usually symptomatic within weeks and therefore rapidly treated in well-resourced settings like Australia [52, 53]. Urethral gonorrhoea has little opportunity to facilitate transmission to other anatomical sites because it is infectious for such a short time [54]. The second major difference is that gonorrhoea very commonly infects the oropharynx, while chlamydia does so infrequently [55–58]. In Australia, oropharyngeal gonorrhoea positivity among MSM attending a sexual health service was around 8% [56] and oropharyngeal chlamydia positivity was around 1% [59, 60]. A case-control study in Seattle-King County estimated that the population attributable risk fraction of urethral gonorrhoea due to insertive penile-oral sex is about 33%, while the population attributable risk fraction of urethral chlamydia due to insertive penile-oral sex is only 3% [61]. A considerable proportion of the sexual practices that are potentially responsible for gonorrhoea transmission involve exposure to the oropharynx, these sexual practices include kissing, oral sex, rimming (oral-anal sex) and the use of saliva as a lubricant during anal sex [48, 62, 63]. Gonorrhoea is also commonly found in expectorated saliva [64]. This was first identified 30 years ago where *N. gonorrhoeae* was cultured from the saliva of 67% of individuals with culture positive oropharyngeal swabs [65]. We recently repeated this work and found that almost half of those (43%) who were culture positive in the oropharynx also were also culture positive in the saliva sample, and all (100%) had a positive result in their saliva by a nucleic acid amplification test (NAAT) [66]. Saliva is commonly used in various sexual activities among MSM and it is our hypothesis that saliva plays a key role in gonorrhoea transmission among MSM [48]. Indeed, kissing has been reported to be the most common sexual behaviour among MSM [67], and gonorrhoea could possibly be transmitted by kissing [48, 62, 68, 69]. Saliva use as a lubricant during anal sex is common among MSM (~70%) [63, 70], and a similar proportion (~70%) of MSM practise rimming [63]. The relatively short duration to spontaneous resolution of untreated oropharyngeal gonorrhoea (<12 weeks) compared to anorectal gonorrhoea (~12 months) [71], means high rates of partner change are required to sustain such a high prevalence of oropharyngeal gonorrhoea [72]. This may be the key reason it is mainly seen in MSM who have large numbers of sexual partners [52].

We hypothesise that it is important to reduce the prevalence and incidence of oropharyngeal gonorrhoea to reduce the overall burden of gonorrhoea [48]. This is because both the prevalence and incidence of gonorrhoea is largely driven by infections at anatomical sites where infections are predominantly asymptomatic, and therefore undetected and untreated, allowing ongoing transmission. In MSM, gonococcal infections at extra-genital sites (i.e. oropharynx and anorectum) are mainly asymptomatic [73–76]. This is also supported by recent mathematical modelling suggesting approximately 75% of incident cases of oropharyngeal gonorrhoea were transmitted from oropharynx to oropharynx through kissing [77], adding weight to another model which suggests gonorrhoea may not be eliminated from the MSM population even with 100% condom-use for anal sex [78]. The implications of these findings for the control of gonorrhoea are clear. If major reductions in gonorrhoea are to be achieved in MSM population, it is essential to reduce gonorrhoea transmission from the oropharynx.

Options for reducing oropharyngeal gonorrhoea cases include use of condoms for oral sex, very frequent screening, antibiotic prophylaxis or use of other agents that could act to prevent the acquisition of infection such as our proposed mouthwash intervention. Given that condom use for anal sex is decreasing [79–81] and MSM rarely use them for oral sex [82], the 3-month average duration of gonorrhoea means that screening would need to be at least every 3 months, as recommended for high-risk Australian MSM [83] but this has been shown to be difficult for MSM to sustain [84]. Antibiotic prophylaxis is unlikely to be adopted because of concerns about emerging resistance. There is no vaccine against gonorrhoea [85]. Thus, mouthwash represents a novel and attractive alternative intervention.

In a recent in vitro study, we showed that 60 seconds of exposure to a commercial alcohol-containing mouthwash product at dilutions of up to 1 in 4, has an inhibitory effect on *N. gonorrhoeae* when assessed at 48 h [86]. In addition, in a small RCT, of 58 MSM who were culture positive for oropharyngeal gonorrhoea, men who were allocated to rinse and gargle the intervention solution (i.e. alcohol-containing mouthwash) for 60 seconds had a significantly lower proportion of positive cultures from the oropharyngeal surface 5 min after use of mouthwash compared to those who were allocated to rinse and gargle the control solution (saline) (52% versus 84%; *p* = 0.013) [86].

### Research hypothesis and aim

The aim of this study is to determine if daily use of antibacterial mouthwash will reduce the incidence of oropharyngeal gonorrhoea in MSM over 12 weeks. We hypothesise that daily use of antibacterial mouthwash could potentially reduce the risk of acquiring oropharyngeal gonorrhoea when used every day and hence reduce the overall incidence of oropharyngeal gonorrhoea.

## Methods/Design

### Trial registration

The trial has been registered on the Australian and New Zealand Clinical Trials Registry (ACTRN12616000247471) on 23rd February 2016.

### Trial design

The OMEGA (Oral Mouthwash use to Eradicate GonorrhoeA) study is a double-blind randomised controlled trial (RCT) of daily use of an antibacterial mouthwash to reduce the risk of acquiring oropharyngeal gonorrhoea in MSM. The trial will be of 12 weeks duration for each participant.

This trial will be conducted within sexual health clinics and high STI-caseload General Practice (GP) clinics in Melbourne and Sydney, Australia. This trial will be conducted in accordance with the guidelines for Good Clinical Practice and reported in accordance with the CONSORT guidelines [87].

### Eligibility criteria for participants

This study is limited to men who have sex with men.

#### Inclusion criteria

- Men who have sex with men and aged 16 years or above, and fulfil either (i) or (ii).
  (i) Men aged 16–24 years: tested positive or negative for oropharyngeal gonorrhoea by NAAT within the previous 30 days, as young MSM are at higher risk of oropharyngeal gonorrhoea [80].
  (ii) Men aged 25 years or above: tested positive for oropharyngeal gonorrhoea by NAAT within the previous 30 days, as this population has a higher rate of repeat diagnosis of gonorrhoea within 90 days [88].
- Provide written informed consent.
- Provide consent for the research team to contact their health professional and/or GP to clarify the use and name of any antibiotics used during the study period.
- Have sufficient English language proficiency to understand the study requirements.
- Men co-infected with HIV or other STIs are eligible.

#### Exclusion criteria

- Travelling (including overseas/interstate) for more than 3 weeks within the next 12 weeks.
- Unable to attend the scheduled week 6 and week 12 visits.
- Report contraindications to mouthwash or food dyes such as allergy.
- Report long-term use (i.e. 4 weeks or more) of antibiotics.
- Not willing to stop using their current mouthwash for the next 12 weeks.
- Previously enrolled into the OMEGA study.
- Men who know someone in their household who is currently enrolled in the OMEGA study. This is to prevent mixing the allocated mouthwashes between participants.
- Individuals who are transgender.

### Trial settings and locations

Participants will be recruited from four large urban publicly-funded sexual health centres in Melbourne and Sydney in Australia: Melbourne Sexual Health Centre, Sydney Sexual Health Centre, Western Sydney Sexual Health Centre, RPA Sexual Health; and one GP clinic that see a high proportion of MSM in Melbourne: Northside Clinic. Potentially eligible participants will be identified by the clinicians and referred to the research nurse who will explain the study and check eligibility using the paper-based eligibility screening template. Eligible participants will be given a participant information sheet and will be asked to provide written information consent prior to commencing the study.

### Intervention

Participants will be randomly allocated to one of two mouthwash solutions. Both are alcohol free commercial mouthwash products that are widely available in supermarkets and pharmacies in Australia – one is commercially advertised to be antibacterial (mouthwash A) and the other is not (mouthwash B). In our in-vitro study, we found that mouthwash A demonstrated an inhibitory effect against *N. gonorrhoeae* on culturing oropharyngeal swabs; this was not the case for mouthwash B.

**Fig. 1:**
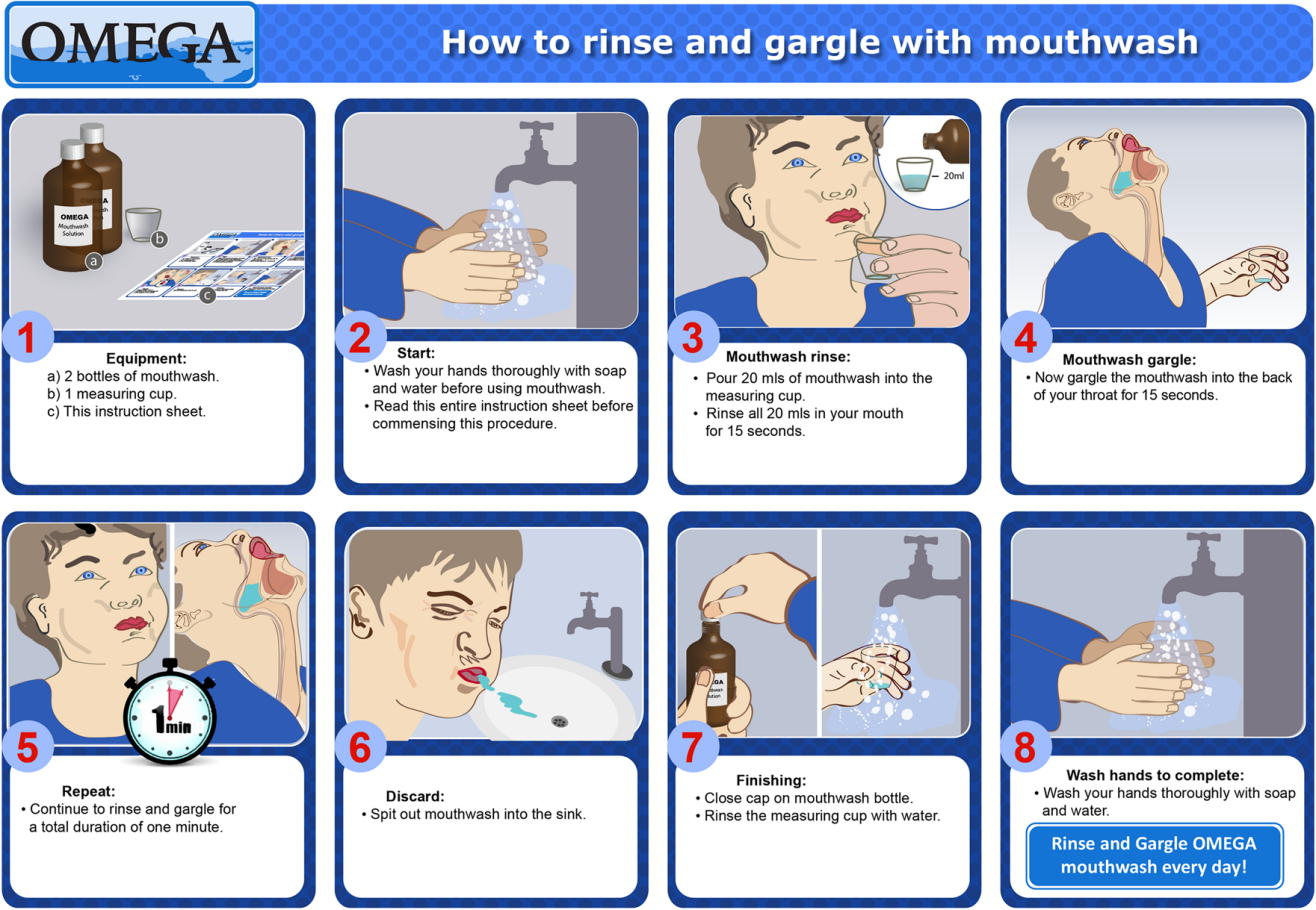
An instruction sheet illustrating the procedure of using the study mouthwash

Participants will receive a total of four study mouthwash bottles (2 × 500 mL bottles at baseline and 2 × 500 mL bottles at week 6) and will be asked to rinse and gargle 20 mL for 60 seconds at least once a day. Rinsing and gargling is important because our previous work has shown that gonorrhoea is detected at both the tonsillar fossae and the posterior oropharynx in MSM [89]. Figure 1 illustrates how to rinse and gargle a mouthwash, and this instruction sheet will be provided to all participants. A video clip is also available on the OMEGA study website for participants to review the procedure (www.mshc.org.au/omega). Participants can use the study mouthwash more than once a day but not more than five times a day. All participants will be required to stop using any other mouthwash they are currently using, during the study period.

#### Extra bottles

All participants will be allowed to request extra bottles at any time between week 0 and week 12. The number of extra bottles given to the participants and reasons will be recorded by the research nurse at each site.

### Outcomes

#### Primary outcome

The primary outcome of this trial is the proportion of MSM diagnosed with oropharyngeal gonorrhoea and the cumulative incidence of oropharyngeal gonorrhoea diagnosed by NAAT at any time point within 12 weeks. We will use NAAT to determine the primary outcome rather than culture because NAAT has a higher sensitivity than culture at the oropharynx [89–91].

#### Secondary outcomes

There are several secondary outcomes in this trial.

1. Proportion of MSM diagnosed with oropharyngeal gonorrhoea by culture within 12 weeks.
2. Adherence to mouthwash over 12 weeks.
3. Acceptability and tolerability of using mouthwash.
4. The proportion of MSM with any of: gonorrhoea at other anatomical sites (urethra and anorectum) or newly acquired chlamydia, HIV or syphilis infection by week 12.

### Sample size

Sample size and power calculations were performed using Stata to compare two proportions (version 13 Stata Corp., College Station, TX, USA) [92, 93]. A previous study has shown that about 10% of MSM had a repeat culture positive for gonorrhoea within 12 weeks after a culture positive for gonorrhoea [88]. With NAAT is a more sensitive test and its detection rate five times higher compared to culture [56], we assumed oropharyngeal gonorrhoea positivity by NAAT in the arm using mouthwash B to be 20% over 12 weeks. Table 1 shows the number of participants required to detect a 50% reduction in oropharyngeal gonorrhoea positivity from 20% in the control arm to 10% in the intervention arm with 5% significance level and 80% and 90% power respectively. Calculations were also based on a 55% and 60% reduction in oropharyngeal gonorrhoea positivity. To detect a 50% reduction in oropharyngeal gonorrhoea positivity with 80% power, a total of 438 men will be required (i.e. 219 in each arm). This is a conservative effect size because our pilot data suggest that a single-dose of mouthwash is associated with a five-fold decrease in detection of gonorrhoea [86]. To allow for up to 15% loss of follow up [94], we will recruit a total of 504 men, 252 in each group. This will ensure 80% power to detect a 50% reduction in oropharyngeal gonorrhoea positivity from 20% to 10%, and 90% power to detect a 50% reduction from 25% to 12.5%.

**Table 1:** Sample size calculations assuming 20% in the control arm will have a positive NAAT for oropharyngeal gonorrhoea within 12 weeks

Efficacy of mouthwash	Oropharyngeal gonorrhoea positivity in the intervention arm	Number of participants	
		80% Power	90% Power
50% reduction	10%	438	572
55% reduction	9%	356	464
60% reduction	8%	294	380

Calculations were based on 5% significance level

### Randomisation

#### Sequence generation

A computer-generated randomisation sequence with a block size of four will be generated using Stata (version 13 Stata Corp., College Station, TX, USA) and held by an independent biostatistician. A 1:1 randomisation ratio, with no stratification, will be used. Enrolled men will be randomised to one of the two arms of the study in equal proportions.

#### Allocation concealment mechanism

The biostatistician will provide the computerised randomisation sequence, containing the name of the mouthwash brand and a study identification number (ID) to two independent staff members at the Melbourne Sexual Health Centre. The two staff members at the Melbourne Sexual Health Centre will have the responsibility for preparing the mouthwash solutions according to the generated randomisation sequence. They will then replace the names of the mouthwash brands with ‘Study mouthwash A’ or ‘Study mouthwash B’ on a new list and provide this list to the principal investigator at each participating site. Only the biostatistician and the two staff members at the Melbourne Sexual Health Centre (who are not the OMEGA study investigators) hold the document containing the code connecting to the real name of the two commercial mouthwashes. Study ID allocation to consenting participants will proceed from the first study number in the randomisation sequence.

### Blinding

**Fig. 2:**
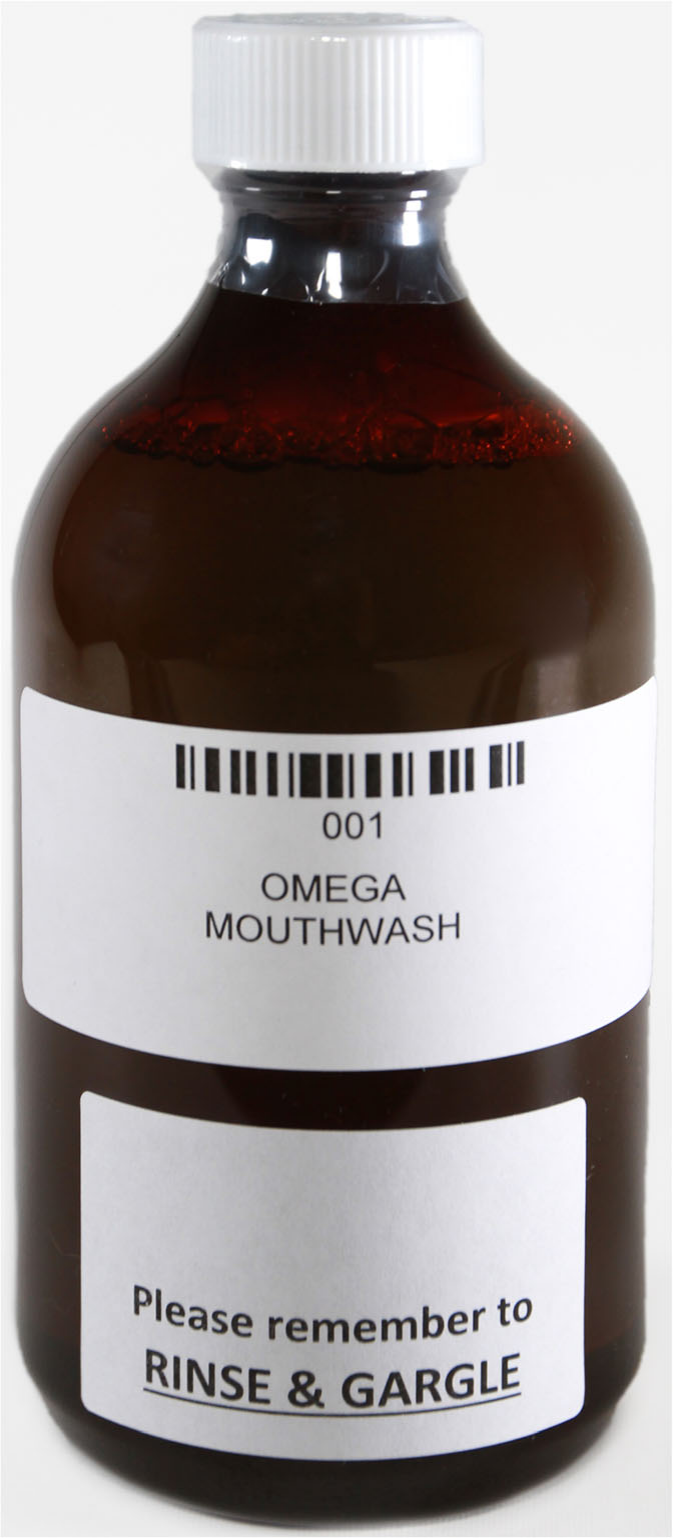
A photograph of the OMEGA mouthwash bottle for a participant, Study ID 001

The study investigators, research nurses and clinicians at each participating clinic, and the participants, are blinded after assignment to interventions. The study mouthwash will be repackaged and numbered at the Melbourne Sexual Health Centre and then sent to the participating clinics. As the two mouthwashes have different packing, colours and tastes according to their respective commercial production, we blinded the products by repacking both mouthwashes into identical containers where the two staff members at the Melbourne Sexual Health Centre will decant the commercial mouthwash into a 500 mL cleaned, opaque amber plastic bottle (Fig. 2). To blind the colour, one drop of food dye will be added to each repackaged bottle to ensure that the two mouthwashes have a similar colour which differs between the original mouthwashes. Since the taste is similar but not identical, we cannot guarantee participant blinding according to taste. However, it will be very unlikely that participants will be aware which mouthwash they use because there are around 70 different commercial mouthwashes available in Australia. The two mouthwash solutions have been tested among clinical staff at the Melbourne Sexual Health Centre and none could distinguish between the two solutions based on colour or taste. The repackaged bottle has a child-resistant cap and protected by a tamper-evident seal. All bottles will be labelled with the study ID in accordance to the randomisation sequence. All procedures (i.e. dispensing, adding food colouring, and sealing) will be performed under sterile conditions. The research nurse will ensure bottles have not been opened before dispensing.

### Trial schema

**Fig. 3:**
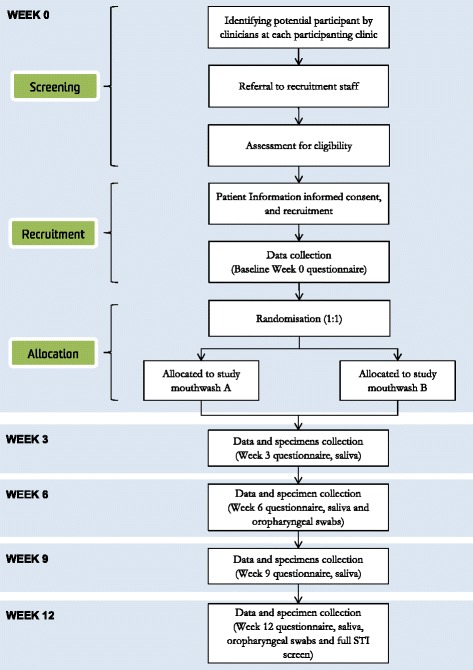
Outline of OMEGA trial schema

Figure 3 outlines the trial schema. This is a 12-week long trial which consists of one baseline clinic visit at week 0, and two follow-up visits at week 6 and week 12. Participants will also be required to return the home-based study pack by post at week 3 and week 9. A reminder SMS will be sent to the participants by the research nurse at each participating site 1 day before the clinic visit and the home-based study pack is due.

#### Baseline

Participants will be asked to complete a questionnaire at the baseline visit will be conducted at the participating clinics.

#### Follow up

Participants will be followed up every 3 weeks during the 12-week trial which consists of two face-to-face clinic visits (at week 6 and 12) and two home-based specimen and data collection postal packs (at week 3 and 9).

**Fig. 4:**
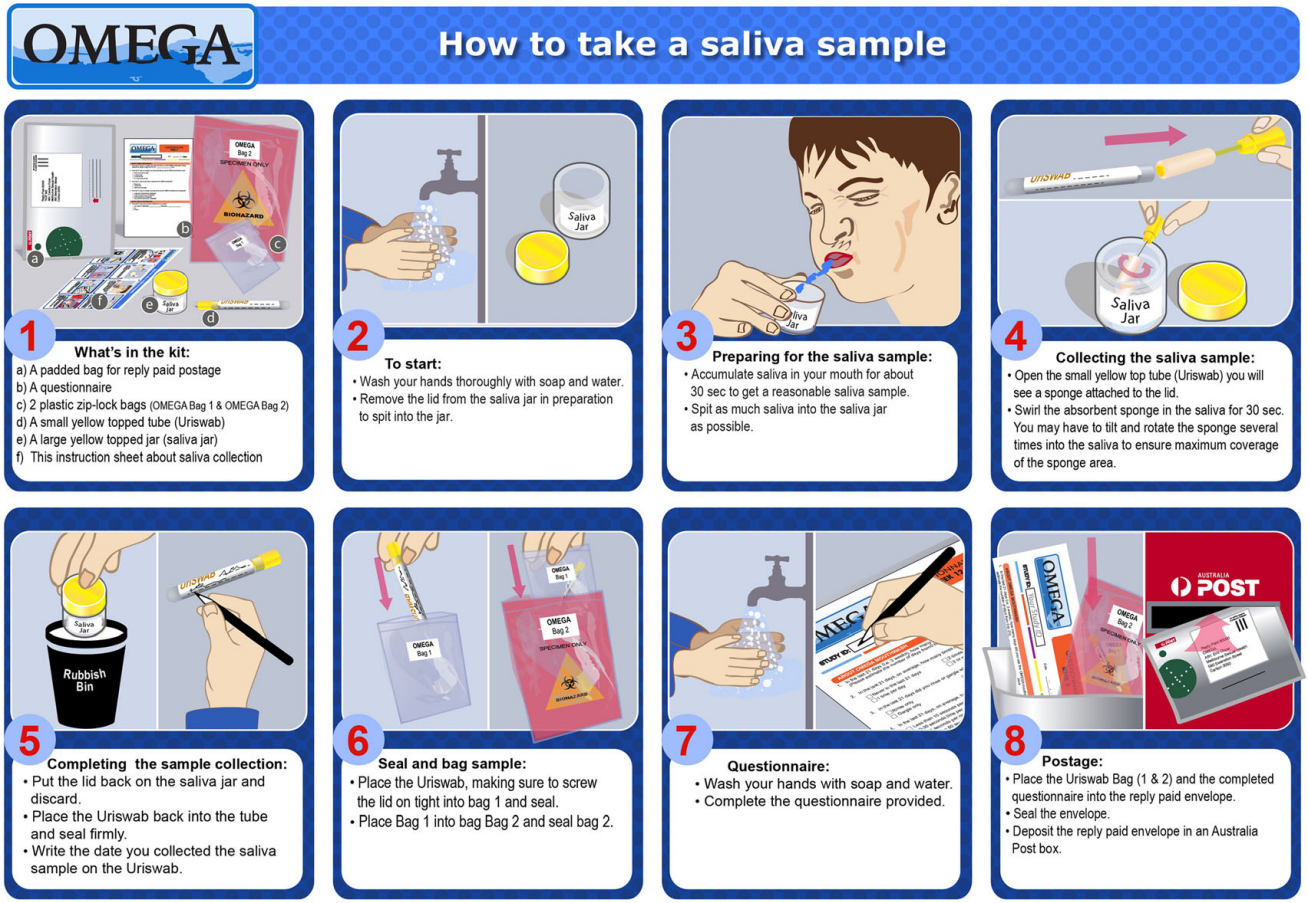
An instruction sheet illustrates the procedure for self-collection of saliva specimens

The week 6 and week 12 follow up visits will be a face-to-face visit and will be conducted at the participating clinics. The follow up time points at week 3 and week 9 will consist of a questionnaire and home-based saliva collection, to be posted back to the Melbourne Sexual Health Centre. The home-based study pack will be disseminated to the participants at both baseline and week 6 clinic visits for week 3 and week 9 home-based study pack, respectively. The home-based study pack consists of: a questionnaire, a yellow cap specimen collection jar, an UriSwab (Copan Diagnostics, Brescia, Italy) for saliva collection, an instruction sheet for saliva collection (Fig. 4) and a prepaid padded postage bag addressed to the Melbourne Sexual Health Centre. All home-based study packs will be returned to the Melbourne Sexual Health Centre only for specimen processing, leaving all clinics to focus on recruitment and clinic visits.

### Biospecimens collection and testing

Biospecimen collection varies depending on the study time point. Table 2 shows the schedule and biospecimens required at each time point.

**Table 2:** Summary of study time and collection of specimens and data

Week	0	3	6	9	12
Location	Clinic	Home	Clinic	Home	Clinic
*A. Biospecimens*					
Oropharyngeal swab on both tonsillar fossae for gonorrhoea tested by NAAT			✓		✓
Oropharyngeal swab on posterior oropharynx for gonorrhoea tested by NAAT			✓		✓
Oropharyngeal swab on both tonsillar fossae for gonorrhoea tested by culture			✓		✓
Oropharyngeal swab on posterior oropharynx for gonorrhoea tested by culture			✓		✓
Oropharyngeal swab on both tonsillar fossae for gonorrhoea and chlamydia tested by NAAT					✓
Oropharyngeal swab on posterior oropharynx for gonorrhoea and chlamydia tested by NAAT					✓
Saliva for gonorrhoea tested by NAAT		✓	✓	✓	✓
First-void urine for gonorrhoea and chlamydia tested by NAAT					✓
Anorectal swab for gonorrhoea and chlamydia tested by NAAT					✓
Serology for HIV and syphilis^a^					✓
*B. Questionnaire data*					
Demographic characteristics (e.g. age, country of birth, education level)	✓				
History of mouthwash use (e.g. frequency, type of mouthwash)	✓				
Sexual risk behaviours	✓	✓	✓	✓	✓
Antibiotic use	✓	✓	✓	✓	✓
Mouthwash adherence		✓	✓	✓	✓
Adverse events		✓	✓	✓	✓
Mouthwash acceptability/tolerability					✓

*NAAT* Nucleic acid amplification test

^a^HIV-positive men will only be tested for syphilis but not HIV

#### Saliva

Participants will be required to self-collect a saliva sample every 3 weeks. Figure 4 illustrates how to self-collect a saliva sample. This instruction sheet will be given to the participants and a video clip is available on the OMEGA study website for participants to review the procedure (www.mshc.org.au/omega). In brief, participants will be instructed to accumulate saliva in the mouth for about 30 seconds to get a reasonable volume of saliva. Participants will spit as much saliva as possible into a yellow cap specimen collection jar, and saliva will be collected by using an UriSwab (Copan Diagnostics, Brescia, Italy). Saliva samples at week 6 and week 12 will be collected at the participating clinic under the research nurse’s supervision; while saliva samples at week 3 and week 9 will be collected at home by participants. All saliva specimens collected will be transported by using UriSwab.

All participants will be required to send their home-based collected UriSwabs to the Melbourne Sexual Health Centre at week 3 and 9 by post. The research nurse at each participating clinic will be required to send clinic-based collected UriSwabs to the Melbourne Sexual Health Centre at week 6 and 12 by post.

Once the research team at the Melbourne Sexual Health Centre receives the UriSwab by post, the UriSwab will be immediately centrifuged at 1200 g for 1 min to separate the saliva from the UriSwab sponge. The centrifuged saliva will be pipetted into a sterile 2.0 mL micro tube (Scientific Specialties, Inc., CA, USA) and stored at −80 °C freezer at the Melbourne Sexual Health Centre for testing.

All centrifuged saliva samples will be batch tested at the Royal Women’s Hospital, Molecular Microbiology Laboratory, Melbourne. The saliva samples will be placed on the MagNA Pure 96 Instrument (Roche Diagnostics, Mannheim, Germany) for DNA purification and will be assessed for sampling and extraction adequacy by amplifying a 260 bp region of the human beta-globin gene [95]. The gonococcal bacterial DNA load of the sample will be quantified using qPCR assays targeting the *N. gonorrhoeae opa* gene. All samples positive for the *opa* gene will then be confirmed using a qPCR targeting the *N. gonorrhoeae porA* gene [96, 97].

#### Oropharyngeal swabs

**Fig. 5:**
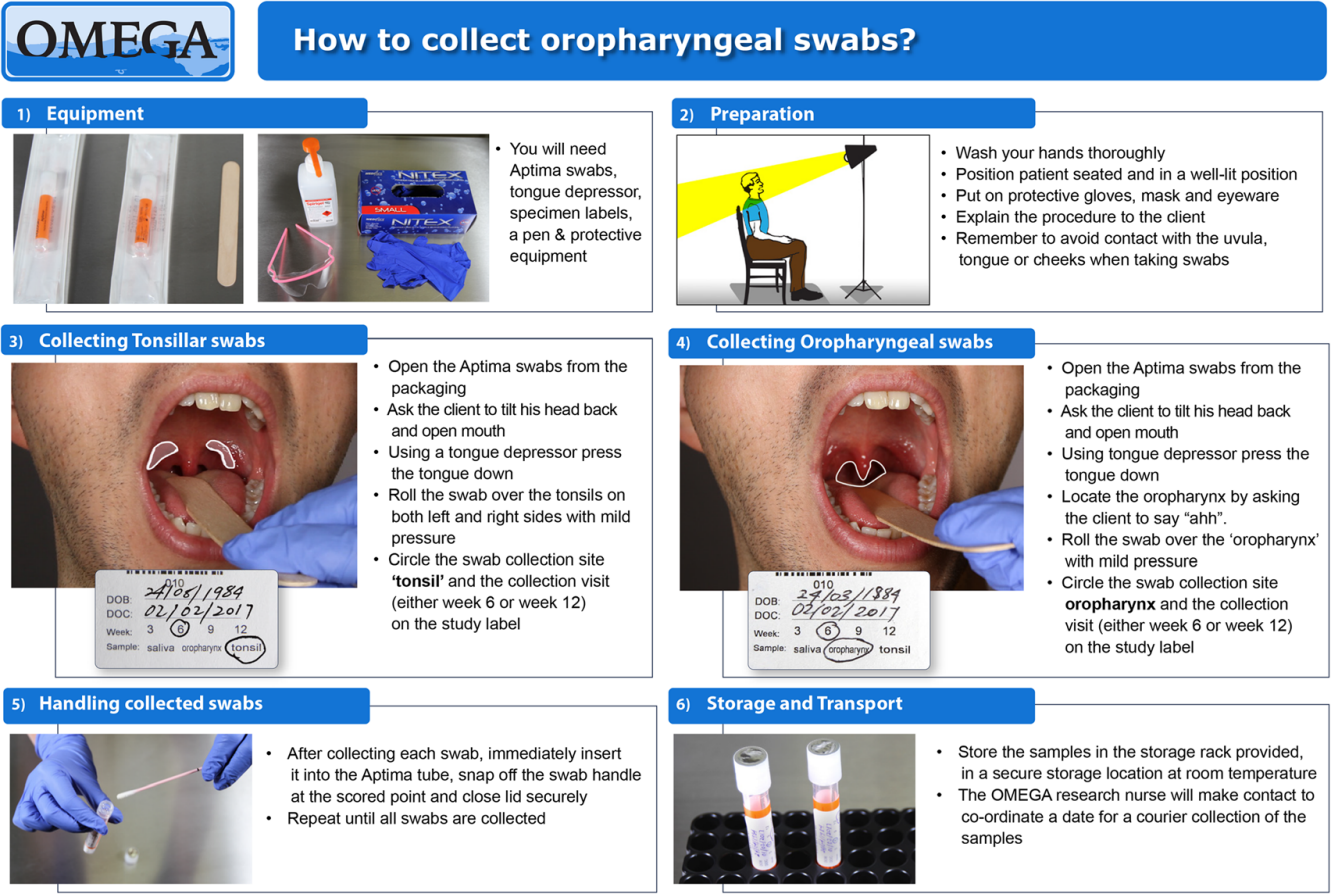
An instruction sheet illustrates the procedure for clinician-collected oropharyngeal swabs

Pharyngeal specimens will be collected at week 6 and week 12 clinic visits. All oropharyngeal swabs will be collected by the research nurses at each participating site. All swabs will be taken by no more than two research nurses at each site to minimise the variability of sampling technique [98]. All research nurses at each site will be trained on how to take a oropharyngeal specimen for this study, and an instruction sheet and a video clip will be provided to the research nurses to review the procedure (Fig. 5).

Six oropharyngeal swabs from the participants will be taken which consists of four standard-of-care specimens and two non-standard-of-care specimens. Table 3 shows the order and type of specimens collected at week 6 and 12 and the corresponding testing methods. Participants will be asked not to use mouthwash on the day of the clinic visit at week 6 and 12 to minimise the effect size if men only use mouthwash at the last minute.

**Table 3:** Summary of oropharyngeal swabs collection at week 6 and week 12 follow up visits

Order of specimens	Type of specimen	Standard-of-care specimens	Testing method	Note
1	Tonsillar fossae	No	All sites: NAAT. The Aptima Combo 2 Assay (Hologic, Inc., CA, USA).	This will be tested in real time as part of stand-of-care specimen for participants recruited at the Melbourne Sexual Health Centre. Thus, swab number 2 is not required for participants recruited at the Melbourne Sexual Health Centre.
2	Tonsillar fossae	Yes	RPA Sexual Health: NAAT. Aptima Combo 2 assay on the Hologic Gen-Probe (San Diego, CA, USA)	Applies to all participating sites except the Melbourne Sexual Health Centre.
			Sydney Sexual Health Centre and Northside clinic: NAAT. Roche cobas 4800 CT/NG (Roche Diagnostic Systems, Branchburg, NJ, USA).	
			Western Sydney Sexual Health Centre: NAAT. BD ProbeTec™ ET System (Becton Dickinson, Sparks, MD, USA).	
3	Posterior oropharynx	No	All sites: NAAT. The Aptima Combo 2 Assay (Hologic, Inc., CA, USA).	This will be tested in real time as part of stand-of-care specimen for participants recruited at the Melbourne Sexual Health Centre. Thus, swab number 4 is not required for participants recruited at the Melbourne Sexual Health Centre.
4	Posterior oropharynx	Yes	RPA Sexual Health: NAAT. Aptima Combo 2 assay on the Hologic Gen-Probe (San Diego, CA, USA)	Applies to all participating sites except the Melbourne Sexual Health Centre.
			Sydney Sexual Health Centre and Northside clinic: NAAT. Roche cobas 4800 CT/NG (Roche Diagnostic Systems, Branchburg, NJ, USA).	
			Western Sydney Sexual Health Centre: NAAT. BD ProbeTec™ ET System (Becton Dickinson, Sparks, MD, USA).	
5	Tonsillar fossae	Yes	All sites: Culture. GC agar plate.	-
6	Posterior oropharynx	Yes	All sites: Culture. GC agar plate.	-

The non-standard-of-care oropharyngeal specimens (swabs number 1 and 3, Table 3) will be collected using cotton-tipped swabs and immediately placed into the Aptima specimen transfer tube containing the transport medium (Hologic, Inc., CA, USA). The Aptima transport medium will be pipetted into a sterile 2.0 mL micro tube and stored at −80 °C until testing. An aliquot of 200 μL of the Aptima transport medium will be placed on the MagNA Pure 96 Instrument (Roche Diagnostics, Mannheim, Germany) for DNA purification. The samples will be assessed for sampling and extraction adequacy by amplifying a 260 bp region of the human beta-globin gene [95]. The *N. gonorrhoeae* bacterial DNA load of the sample will be quantified using qPCR assays targeting the *N. gonorrhoeae opa* gene. All positive samples for the gonococcal *opa* gene will then be confirmed using a qPCR targeting the *N. gonorrhoeae porA* gene [96, 97]. All non-standard-of-care oropharyngeal specimens (swabs number 1 and 3, Table 3) will be batch tested at the Royal Women’s Hospital, Molecular Microbiology Laboratory, Melbourne.

The standard-of-care oropharyngeal specimens (swabs number 2, 4, 5 and 6, Table 3) will be undertaken locally as part of routine care at each participating clinic. Swabs number 2 and 4 will be tested by in-house NAAT methods. The Melbourne Sexual Health Centre, and RPA Sexual Health use the Aptima Combo 2 assay on the Hologic Gen-Probe (San Diego, CA, USA) PANTHER system. The Western Sydney Sexual Health Centre uses the strand displacement amplification (SDA) assay on the BD ProbeTec™ ET System (Becton Dickinson, Sparks, MD, USA). The Sydney Sexual Health Centre and the Northside Clinic use real-time polymerase chain reaction (PCR) on the Roche cobas 4800 CT/NG (Roche Diagnostic Systems, Branchburg, NJ, USA). These assays have been shown to have analytical sensitivity and specificity of 100% [99, 100]. Test results for oropharyngeal gonorrhoea at week 6 and week 12 will be given to the participants. Participants with a positive test will be recalled to the clinic for standard antibiotic treatment as per the Australian STI treatment guidelines [101]. Swabs 5 and 6 will be tested by in-house culture method. Results for standard-of-care oropharyngeal specimens will be recorded.

#### Full STI screening at week 12

All participants will have a STI screen at week 12 as per the Australian STI guidelines [102]. This includes oropharyngeal swabs for chlamydia by NAAT, first-void urine for urethral gonorrhoea and chlamydia by NAAT, anorectal swab for gonorrhoea and chlamydia by NAAT, and serology for HIV and syphilis. All specimens will be collected locally and will be tested by in-house methods at each participating clinic. Test results will be given to the participants. Participants with a positive test will be recalled to the clinic for standard antibiotic treatment as per the Australian STI treatment guidelines [101].

### Data collection

#### Questionnaire

Participants will be required to complete a self-administered questionnaire at baseline and at week 6 and week 12 at the clinic (Table 2). Participants will also be asked to complete a short questionnaire at week 3 and week 9 and return it by post. These questionnaires collect information on demographic characteristics (at baseline), history of mouthwash use (at baseline), antibiotic use, sexual risk behaviours, mouthwash adherence (after baseline), adverse events (after baseline) and mouthwash acceptability/tolerability (at week 12).

#### Mouthwash adherence monitoring

All five questionnaires will ask about mouthwash adherence. Participants will be required to report the number of days they use the OMEGA study mouthwash, how many times they use it per day, the volume they use each time, how they use it (i.e. rinse only; gargle only; both rinse and gargle) and the duration of the gargle/rinse each time. Participants will also be required to return the mouthwash bottles to the clinic at week 6 and 12. The volume left in the mouthwash bottle will be measured and recorded by the research nurse. High mouthwash adherence over a 2-week period has been reported among 10 MSM [103].

#### Antibiotic use

All five questionnaires will ask about use of antibiotics. If the participants report any antibiotic use, the name of the antibiotic and reason for use will also be asked. In signing the consent form, participants also consent to the research team contacting their general practitioner and other health professionals at their local clinic(s) to obtain the information on antibiotic use (i.e. date of prescription, name of antibiotics) and gonorrhoea diagnoses during the 12-week study period. A cover letter and data request form will be sent to the GP by the research nurse once the participants have completed the study.

### Treatment

Participants with any STI during the study period will be treated as per the Australian STI guidelines [83]. Any antibiotic given to the participants will be recorded.

### Adverse events

Adverse event and tolerability data will be measured from the questionnaires every 3 weeks. The Medical Dictionary for Regulatory Activities (MedDRA) will be used for reporting any adverse events. No adverse events were reported from a previous longitudinal study involving 10 men who used mouthwash every day for 14 days [103].

### Loss to follow-up

Participants will be considered ‘lost to follow up’ if they do not attend for the week 12 clinic visit by 14 weeks since the baseline visit.

### Compensation

Reimbursement is necessary to compensate participants for their participation in the trial and to maximize retention. Participants will receive an AU$100 gift voucher when they attend the clinic in person at week 12. Similar reimbursements have been used in a cohort study among MSM with four clinic visits with 85% retention at 12 months [104].

### Statistical methods

Data will be analysed in Stata (version 13 Stata Corp., College Station, TX, USA). The primary analysis will be an intention to treat analysis for all those with at least one follow up swab. The demographic characteristics and sexual risk behaviours will be compared between the control and intervention arm to ensure the balance in the baseline characteristics in both arms [105].

#### Primary analysis

The primary end-point will be the proportion of men who have gonorrhoea detected in the oropharynx by NAAT at any time within 12 weeks in any specimen (saliva and/or oropharyngeal swabs). The proportion of men with oropharyngeal gonorrhoea detected will be compared between both groups. The 95% confidence intervals (CI) of the proportion of men with oropharyngeal gonorrhoea will be calculated based on the ‘exact’ binomial confidence intervals [106]. The two study arms will be compared using logistic regression. Multivariate logistic regression will be performed to adjust any imbalance of baseline characteristics and potential confounding factors. Men with symptomatic urethral gonorrhoea may attend clinical services between the two clinic visits and test positive for oropharyngeal gonorrhoea. Any positive oropharyngeal results will be included in the analysis unless they were taken within 3 weeks of treatment because within 3 weeks NAAT results may represent residual non-viable DNA from the previous infection [107].

#### Secondary analysis

The cumulative incidence of oropharyngeal gonorrhoea by week 12 will also be calculated. Treatment arms will be compared using survival analysis, clustered by each individual to allow for multiple events. Individuals who tested positive for oropharyngeal gonorrhoea during the study period will be treated and remain in the study until end of week 12. Individuals will contribute at least two time periods of follow-up (i.e. time between each test).

The proportion of men who have gonorrhoea detected in the oropharynx by culture within 12 weeks will also be calculated as a secondary outcome, the statistical approach will be the same used for the primary analysis. A sensitivity analysis will also be performed comparing treatment arms in terms of gonorrhoea detection at 6 or 12 weeks using repeated measures logistic regression methods.

The adherence to mouthwash over 12 weeks will be collected from the questionnaire every 3 weeks. The number of days using mouthwash over the 12-week period will be calculated for each participant, and stratified by treatment arm.

Acceptability and tolerability of using mouthwash over 12 weeks will be measured. Participants will be asked about any discomfort and difficulties in using the mouthwash every 3 weeks.

The proportion of men who have other STIs (i.e. gonorrhoea at other site except at the oropharynx, chlamydia, HIV and syphilis) by 12 weeks will also be calculated as a secondary outcome and stratified by treatment arm.

### Trial status

The trial commenced recruitment in March 2016 at the Melbourne Sexual Health Centre. It is expected recruitment will commence at other sites (Sydney Sexual Health Centre, Western Sydney Sexual Health Centre, RPA Sexual Health, Northside Clinic) in June 2017. It is expected this trial will be completed by December 2018.

### Ethical consideration

This study protocol has been approved by the Alfred Hospital Ethics Committee in Melbourne, Victoria (project number 29/16; HREC/17/Alfred/13).

## Discussion

The rising rates of gonorrhoea in MSM urgently require novel, safe and effective non-condom-based and non-antibiotic-based interventions to reduce the incidence of gonorrhoea that are not associated with generation of antibiotic resistance. Epidemiological, clinical and mathematical modelling data provide substantial evidence that the oropharynx is the key driver of gonorrhoea in MSM [52]. Recent laboratory data and an RCT have shown that a single-dose of mouthwash has a short-term inhibitory effect against *N*. *gonorrhoeae* in the oropharynx [86]. However, it is unclear whether daily use of mouthwash can prevent acquisition of oropharyngeal gonorrhoea and this will be the first multicentre RCT to evaluate whether daily use of mouthwash could reduce the risk of oropharyngeal gonorrhoea acquisition. If we show daily use of mouthwash is effective in reducing the prevalence and incidence of oropharyngeal gonorrhoea, this will be the first acceptable, widely available and easily implementable non-drug intervention for gonorrhoea control since the widespread use of condoms. Mouthwash is commonly used albeit not often daily [108], and it therefore has the potential to rapidly be implemented in the community. If this intervention is proven to be effective it will change national and international strategies for gonorrhoea prevention and control.

We have limited this trial to MSM only because oropharyngeal gonorrhoea is rare in transgender individuals and heterosexuals and transmission between men and women may less frequently involve the oropharynx [109–112]. The only group of heterosexuals where oropharyngeal transmission may play a significant role is female sex workers, particularly in Asian countries [113–115].

There has previously been some controversy about the use of mouthwash and increased the risk of oral cancer [116–118]. However, a review and a meta-analysis of epidemiological studies have shown there is no association between oral cancer and mouthwash use (including daily use, and alcohol-containing mouthwash) [119, 120]. This is therefore anticipated to be a completely safe intervention.

The aim of daily use of mouthwash is to reduce the prevalence and incidence of gonorrhoea infection in the oropharynx in order to reduce further ongoing transmission to their partners, but not as an alternative treatment for gonorrhoea. A recent modelling study has estimated that there would be a three-fold reduction in prevalence of oropharyngeal gonorrhoea if 50% of men use the mouthwash on 50% of days. In addition, if 75% of men use the mouthwash on 75% of days, it may achieve a scenario close to elimination from MSM populations [77].

Topical antiseptics have been used against STIs for more than 100 years, but only for genital infections [121]. This trial will be the first study to evaluate the role of topical antiseptics used against STIs other than genital infections. If we show that antibacterial mouthwash reduces oropharyngeal gonorrhoea infection, then these findings could lead to a widespread public health intervention to increase mouthwash use among sexually active MSM. The ultimate outcome may be a substantial fall in gonorrhoea among MSM and a reduced probability of new resistant gonococcal strains emerging.

## Abbreviations

HIV: Human immunodeficiency virus
HREC: Human Research Ethics Committee
MSM: Men who have sex with men
NAAT: Nucleic acid amplification test
NHMRC: National Human and Medical Research Council
OMEGA: Oral Mouthwash use to Eradicate GonorrhoeA
PrEP: Pre-exposure prophylaxis
RCT: Randomised controlled trials
SMS: Short message service
STI: Sexually transmissible infection
